# Malignant Vascular Tumors of the Head and Neck—Which Type of Therapy Works Best?

**DOI:** 10.3390/cancers13246201

**Published:** 2021-12-09

**Authors:** Susanne Wiegand, Andreas Dietz, Gunnar Wichmann

**Affiliations:** Department of Otolaryngology, Head and Neck Surgery, University Hospital Leipzig, 04103 Leipzig, Germany; Andreas.Dietz@medizin.uni-leipzig.de (A.D.); Gunnar.Wichmann@medizin.uni-leipzig.de (G.W.)

**Keywords:** angiosarcoma, epithelioid hemangioendothelioma, head neck cancer

## Abstract

**Simple Summary:**

Malignant vascular tumors are extremely rare tumors with variable clinical courses, and few data on their clinical management are available. Diagnosis is difficult due to their wide morphologic appearance. The intent of the present review is to demonstrate the current knowledge and management on malignant vascular tumors of the head and neck area. The mainstay of treatment for malignant vascular tumors is surgery, but radiotherapy and chemotherapy are also parts of the treatment concept especially in angiosarcomas. Targeted therapy, antiangiogenetic drugs and immunotherapy have been studied as new treatment options.

**Abstract:**

Malignant vascular tumors of the head and neck are rare neoplasms with variable clinical presentation, wide age distribution, and variable clinical courses. The heterogeneous presentation of angiosarcomas and epithelioid hemangioendothelioma often leads to misdiagnosis and unsuitable treatment. While risk factors for angiosarcomas are previous radiation, chronic lymphedema, and exposure to arsenic, thorium oxide, or vinyl chloride, there are only limited and retrospective data available on prognostic factors in EHE. In both angiosarcomas and EHE, surgery is the mainstay of treatment. There is limited evidence regarding the role of radiotherapy in EHE, although EHE is considered relatively radiosensitive. In angiosarcomas, adjuvant radiotherapy is recommended according to retrospective case series. A standard medical therapy for metastasized malignant vascular tumors is lacking. Chemotherapy, which is effective in angiosarcoma, is mostly ineffective in EHE. Targeted therapy, antiangiogenetic drugs and immunotherapy have been studied as new treatment options. The goal of this review is to summarize the current data regarding malignant vascular tumors along with their diagnosis and management.

## 1. Introduction

Malignant vascular tumors are a part of the spectrum of vascular anomalies. The classification of the International Society for the Study of Vascular Anomalies (ISSVA) differentiates vascular anomalies into vascular tumors and vascular malformations. Vascular tumors are further subdivided into benign vascular tumors (of which infantile hemangioma is the most common), locally aggressive or borderline vascular tumors, and malignant vascular tumors (see Table 1).

Malignant vascular tumors pose a diagnostic and therapeutic challenge. Due to their low incidence, there is insufficient clinical awareness. The rarity of angiosarcoma and epithelioid hemangioendothelioma combined with their wide variety of symptoms and age distribution at presentation, different anatomic sites, and variable clinical courses lead to a wide range of differential diagnoses. Reliable data about malignant vascular tumors are difficult to obtain, as much of the information on these tumors is obtained from retrospective surveys.

Using data from the National Cancer Institute’s Surveillance, Epidemiology, and End Results program 1973 to 2012, 1394 angiosarcoma and 221 EHE cases were evaluated in 2016. Most patients were white (85%), followed by African American (7%), without differences in incidence according to gender. The mean age at the time of diagnosis was 63 years. Survival was better in younger patients, with an overall 5-year relative survival rate in patients <50 years of 41.9%, and of 18.2% in patients ≥50 years. Patients with angiosarcoma had a 5-year survival rate of 24.5%, while patients with EHE had a 5-year survival rate of 41.9%. Multilocular disease was detected in almost 30% of patients with angiosarcoma, compared to 16.3% of patients with EHE. Surgical resection was the mainstay of treatment (80%), and was associated with improved 5-year survival in patients with higher-grade tumors [2]. Although survival rates are poor, there are no specific treatment guidelines for malignant vascular tumors except that the guidelines of the National Comprehensive Cancer Network (NCCN) and European Society for Medical Oncology (ESMO) for soft-tissue sarcomas also apply for angiosarcoma and epithelioid hemangioendothelioma.

The present review should demonstrate the present knowledge and management of malignant vascular tumors of the head and neck area.

## 2. Angiosarcomas

Angiosarcomas are malignant endothelial cell tumors of vascular or lymphatic origin with variable clinical presentation. They may arise in any part of the body, but about 50% of all angiosarcomas involve the skin of the head and neck [3]. Men are affected more than women, with a peak age of incidence in the seventh decade [4]. Angiosarcomas arise from endothelial cells of blood or lymph vessels either sporadically or secondary to prior radiation therapy. In these cases, the breast is the most affected site [5]. Other risk factors seem to be chronic lymphedema and exposure to arsenic, thorium oxide (Thorotrast), or vinyl chloride, where hepatic angiosarcomas especially arise, as well as familial syndromes like Neurofibromatois Nf-1, mutated BRCA1 or BRCA2, Maffucii syndrome, and Klippel–Trenauney syndrome [6].

### 2.1. Clinical Presentation

Diagnosing angiosarcoma remains a challenge due to the non-specific and variable clinical presentation. The diagnosis is often delayed by its apparently benign clinical appearance, which can be confused with a skin infection or soft tissue trauma [3,5]. A delay in the diagnosis of angiosarcoma can affect survival. Clinical suspicion and prompt diagnosis are therefore essential for successful initiation of therapy.

**Figure 1:**
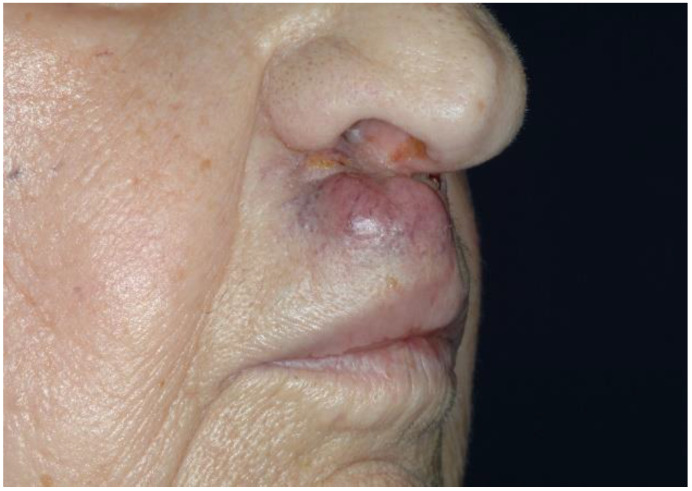
Cutaneous angiosarcoma.

Cutaneous angiosarcoma usually presents as bruise-like purpura or a raised purplish-red papule that has been present for several months and can be rapidly growing and may be associated with ulceration and hemorrhage [4] (Figure 1).

Cutaneous angiosarcoma could be classified into different types. Primary cutaneous angiosarcoma originating in the absence of previous irradiation or lymphedema usually affects the scalp or frontal region of elderly men. Angiosarcoma based on chronic lymphedema occurs predominantly in the upper limbs of women after radical mastectomy and axillar dissection, but can also be seen in patients with congenital, traumatic, or infectious lymphedema. Post-radiation angiosarcoma is also observed with greater frequency in women, on average 5–10 years after radiotherapy for treatment of breast carcinoma, and is currently the second most frequent subtype. Angiosarcomas of the soft tissue or abdominal organs typically present as expanding masses associated with pain or discomfort. Due to hematogenous spread, the lungs are the most common site for angiosarcoma metastases, followed by brain, liver, bone, soft-tissue structures, and lymph nodes. About 50–80% of patients with angiosarcomas present with localized disease; metastatic disease at presentation is estimated to occur in 20–45% [7,8,9].

### 2.2. Staging

Depending on the localization, there are different staging systems for angiosarcomas of the head and neck, trunk and extremities, abdomen and thoracic visceral organs, and retroperitoneum. The diagnostic work-up for angiosarcomas of the head and neck should include an MRI or CT scan and a chest, abdominal and pelvic CT. Depending on the localization, CNS imaging with MRI should also be considered. PET/CT scan may also be useful [10]. On contrast-enhanced CT, angiosarcoma typically manifests as an irregular enhancing soft tissue mass. On MRI, angiosarcomas show intermediate signal intensity on T1-weighted images and high signal intensity on T2-weighted magnetic resonance images, with aggressive infiltration of adjacent tissues. There can be areas of high signal intensity representing hemorrhage on T1-weighted images. Within the tumors, the signal intensity of vascular structures may reflect high flow (low signal intensity on all MR images, regardless of pulse sequence) or low flow (high signal intensity on T2-weighted images). After the administration of intravenous gadolinium-based contrast material, angiosarcomas enhance and may show central areas of necrosis [11]. Several studies have previously demonstrated that 18F-FDG PET/CT is a valuable method for staging, predicting prognosis and evaluating the therapy response of angiosarcoma [12,13]. According to the results of a study analyzing the prognostic value of FDG PET/CT parameters in the evaluation of patients with head and neck soft-tissue sarcomas of various subtypes, maximum standardized uptake value (SUV_max_), metabolic tumor volume (MTV), and total lesion glycolysis (TLG) were significantly associated with disease-specific and overall survival. Patients with a tumor SUV_max_ value of >7.0 experienced an approximately fivefold increase in mortality in terms of survival relative to those with a tumor SUV_max_ < 7.0 [12]. In patients with angiosarcoma of different locations it also could be demonstrated that higher pSUVmax, MTV, whole-body TLG, and whole-body TLG ratio correlated significantly with poorer overall survival [13].

### 2.3. Histology

Angiosarcoma has a wide morphologic appearance, ranging from lesions that are cytologically bland and vasoformative to solid sheets of highly pleomorphic cells without definitive vasoformation [4]. Because of the heterogeneous histologic features in poorly differentiated tumors, the histological identification of an angiosarcoma can be challenging. Angiosarcoma cells are typically plump, pleomorphic and mitotically active. They can be spindle-shaped, polygonal, epithelioid, or round, with some forming papillae or solid nests within vascular lumina [4].

In cutaneous angiosarcoma, tumor vessels ramify the dermis and intercalate through dermal collagen and subcutaneous soft tissues. Intratumoral hemorrhage is common. Although no immunohistochemical staining is pathognomonic, angiosarcomas express typical vascular markers like CD31, CD34, Fli1 and ERG and occasionally podoplanin (D2-40). The morphological appearance of head and neck cutaneous angiosarcoma is somewhat distinguished from other locations by its common association with a heavy lymphocytic infiltrate. The tumors typically consist of highly infiltrative vascular proliferation lined with relatively uniform cells, with sparse cytoplasm and small but hyperchromatic nuclei. The diagnosis can be difficult due to its partly minor characteristics of malignancy and the dense inflammatory infiltrate. Until now, no specific molecular abnormalities have been associated with this subset [4].

Recently, mutations and amplifications have been described for angiosarcoma; most of these aberrations occur in the tyrosine-kinase pathways specific for vascular receptors. PTPRB, PLCG1, CIC, KDR, and FLT4 mutations and MYC amplifications have been described for angiosarcoma [4]. In contrast to other sarcomas, angiosarcoma shows a very low level of alterations in the p53 and PIK3CA/AKT/mTOR pathways, and no PTEN alterations were identified in a series of primary and secondary angiosarcoma samples [14].

### 2.4. Treatment

#### 2.4.1. Localized Disease

Though there are retrospective case series, there are only a few prospective trials analyzing angiosarcoma treatment, and randomized trials are lacking. Therefore, no evidence-based recommendations can be made for angiosarcomas of the head and neck.

Surgical resection is the mainstay of treatment, and R0 resection with wide margins is the only curative modality for localized disease. Achieving negative margins during surgery is often difficult due to the invasive and often multifocal growth and the close relationship to important anatomical structures of the head and neck region. In case of positive resection margins on final pathology, surgical re-resection to obtain negative margins should be performed if possible [10], as positive margins worsen the prognosis [5].

Because of the high risk of local recurrence, adjuvant radiotherapy is recommended according to retrospective case series, although no formal radiotherapy trials have been done. In many studies, improved local control and overall survival after adjuvant radiotherapy could be demonstrated [15,16]. In patients with cutaneous angiosarcoma of the face/scalp treated curatively with surgery, RT, or a combination of surgery and RT, Guadagnolo et al. demonstrated that combined-modality therapy (vs. surgery alone or RT alone) was associated with improved overall survival (OS), disease-specific survival (DSS), and local control. The OS rate was 43% at 5 years, and DSS was 46% at 5 years. Tumor size >5 cm and satellitosis were prognostic for inferior OS and DSS [17]. In a study of 48 patients with localized angiosarcoma of the scalp and face, patients treated with both surgery and radiotherapy (2-year OS: 45.8%) had a significantly more favorable OS (*p* < 0.0001) than patients treated with either surgery or radiotherapy (2-year OS: 11.1%) or patients treated with neither surgery nor radiotherapy (2-year OS: 0%) [18].

Adjuvant radiotherapy is recommended with high doses (>50 Gy) and wide treatment fields [19]. Scott et al. recommended at least 60–65 Gy for the postoperative tumor bed and 70–75 Gy for patients who receive radiation monotherapy [20].

Other trials failed to identify a benefit of adjuvant radiotherapy [8,21]. In a meta-analysis of cutaneous angiosarcoma, the 5-year survival rates in patients were 12.5–46.9% after surgery and 0–16.7% after radiotherapy. Surgical treatment had the highest 3-year survival rate; however, as the follow-up time was extended, the survival rate decreased, especially from 3-year to 5-year: for surgery, from 60.2% to 12.5–46.9%; for RT, from 33.3% to 0–16.7%; for surgery and RT, from 58.4% to 0–33.3% [21]. Radiotherapy alone is generally thought to be an inadequate treatment for potentially curable disease [5]; therefore, definitive RT should only be performed in unresectable cases. According to a series of investigations, higher doses (>70 Gy) have been found to potentially improve local control and OS when treating with radiotherapy alone [22,23].

#### 2.4.2. Metastatic Disease

Cytotoxic chemotherapy is the primary treatment option for metastatic angiosarcoma. The mainstay of cytotoxic chemotherapy drugs for angiosarcomas consists of anthracycline, ifosfamide and taxanes (paclitaxel and docetaxel) [5]. An overview of systemic therapy for angiosarcoma is shown in Table 2.

Angiosarcoma response and survival following first-line anthracycline-based chemotherapy seems to be similar to other soft tissue sarcoma histotypes [5]. Pooled data from eleven prospective randomized and non-randomized European Organisation for Research and Treatment of Cancer (EORTC) clinical trials of first-line anthracycline-based chemotherapy for advanced soft tissue sarcomas demonstrated a median PFS of 4.9 months and OS of 9.9 months for angiosarcomas [36]. The combination of doxorubicin and ifosfamide was associated with improved PFS (HR 0.53, 95% CI 0.33–0.86; *p* = 0.010) and OS (HR 0.53, 95% CI 0.32–0.90; *p* = 0.018) compared to single agent anthracyclines [36].

A phase I/II study of docetaxel, ifosfamide, and doxorubicin in advanced, recurrent, or metastatic soft tissue sarcoma showed that ifosfamide combined with either doxorubicin or docetaxel both had the same response, but better overall survival at 17 months [43]. In a trial comparing doxorubicin and weekly paclitaxel for metastatic angiosarcomas, Italiano et al. demonstrated that weekly paclitaxel seemed to have similar efficacy to doxorubicin [34].

The optimal sequence of anthracycline and taxane-based chemotherapy remains unclear. Some studies suggest higher responses to taxanes in head/neck cutaneous angiosarcomas [16,24]. Until now, no prospective randomized trials have been performed to compare different chemotherapy regimens in angiosarcoma.

Targeted therapy in patients with angiosarcomas focuses on vascular endothelial growth factor A and tyrosine kinase, and includes drugs like imatinib, sorafenib, pazopanib, and bevacizumab. In the clinical trials, imatinib, sorafenib, and bevacizumab showed limited efficacy against angiosarcomas while increasing toxicity, especially when combined with paclitaxel [30,33]. Pazopanib activity in angiosarcomas is comparable to other soft tissue sarcomas. A phase III trial on pazopanib for metastatic soft tissue sarcomas progressing on at least one anthracycline-containing regimen demonstrated a PFS of 4.6 months, although there was no OS benefit [44]. In a study of pazopanib as a second or later line of therapy, a progression-free survival of 3 months and an overall survival of 9.9 months were reported [39]. In other studies, similar results were obtained [45,46].

Propranolol has been tested in combination with other chemotherapeutic agents such as vinblastine and cyclophosphamide [47]; however, evidence is lacking to make any recommendations regarding the use of propranolol for treatment of patients with angiosarcoma.

Recently, programmed death ligand-1 (PD-L1) expression was shown to be inversely correlated with the prognosis of patients with cutaneous angiosarcoma [48].

In a prospective phase II trial, PD-1 inhibition had limited activity in 57 patients with soft tissue sarcomas, including one patient with angiosarcoma [49]. In a cohort of patients with metastatic or unresectable angiosarcoma, dual anti-CTLA-4 and anti PD-1- blockade demonstrated an overall response rate of 25%, with three of five patients with cutaneous tumors of the scalp or face responding to treatment [42].

These data demonstrate that a standard systemic therapy is still lacking in angiosarcoma, and larger research efforts to clarify the role of drug therapy in angiosarcoma are urgently needed.

## 3. Epithelioid Hemangioendothelioma

Epithelioid hemangioendothelioma is a rare vascular tumor with an estimated prevalence of less than one in one million [50]. The term EHE was introduced in 1982 by Weiss and Enzinger, referring to a vascular tumor of bone and soft tissue showing features between hemangioma and angiosarcoma [51].

EHEs of the head and neck are uncommon [52]; most primaries are located in the liver, lung, and bone, but cases in the breast, lymph nodes, and other soft tissues have also been reported [53]. Regarding the localization in the head and neck, there have been cases described in various areas including the oral cavity (gingiva, palate, floor of mouth), parotid gland, vocal fold, nasal cavity, and thyroid [54,55,56,57,58]. Metastases from other primary sites can also involve the neck [57].

In 2013, EHE was classified as a locally aggressive tumor with metastatic potential by the World Health Organization (WHO) [59]. It usually affects middle-aged persons, but age at diagnosis ranges from childhood to high age. Although EHE progresses slowly, the reported incidence of metastases is 20–30% and mortality is 15% [59]. The mean survival time after diagnosis is 4.6 years. Poor prognosticators were male sex, age above 55 years, presence of pulmonary lesions, and multi-organ involvement [60]. Using an internet registry to identify clinical patterns with prognostic significance in EHE, Lau et al. identified the following clinical features to be strongly correlated with reduced survival: signs of uncontained spread such as pleural effusion or ascites, hemoptysis, and tumor in three or more bones [61].

### 3.1. Etiology

A hallmark molecular characteristic of EHE is a recurrent t(1;3) translocation resulting in a WWTR1-CAMTA1 fusion gene, which is present in 90% of EHE cases and pathognomonic for disease [62,63]. This recurrent translocation has not been detected in any of the morphologic mimics of EHE, and can therefore serve as a molecular diagnostic tool in challenging cases. However, other genetic alterations have also been described in EHE. In a WWTR1-CAMTA1 fusion-negative subset of patients characterized by a somewhat different morphology, including focally well-formed vasoformative features, Antonescu et al. detected YAP1-TFE3 fusions. This genetic alteration seems to arise mainly in young patients [64].

In a study of 49 participants with EHE and *WWTR1-CAMTA1* fusion, more than half of the patients exhibited a secondary genomic variant. Advanced stage (III/IV) EHE and older age (>45 years) was especially strongly associated with the presence of pathogenic secondary genomic variants. The most prevalent gene alteration was deletion of the *CDKN2A/B* locus, corresponding to well-studied tumor suppressor genes responsible for regulation of the cell cycle and p53-mediated apoptosis. Other commonly altered genes included *RB1*, *APC*, and *FANCA*. Up to 20% of the genetic alterations are potentially clinically actionable [63].

Another hypothesis regarding the pathogenesis of EHE refers to a causal relationship between chronic *Bartonella* infection and tumor development, as Mascarelli et al. detected *Bartonella* bacteremia in two patients with EHE [65]. *Bartonella* is the only bacterial genus known to cause endothelial proliferation, presumably by inducing aberrant angiogenic VEGF signaling analogous to the angiogenic pathogenesis in malignant tumors. These results suggest that Bartonella may play a role the development of vascular tumors.

### 3.2. Clinical Presentation

**Figure 2:**
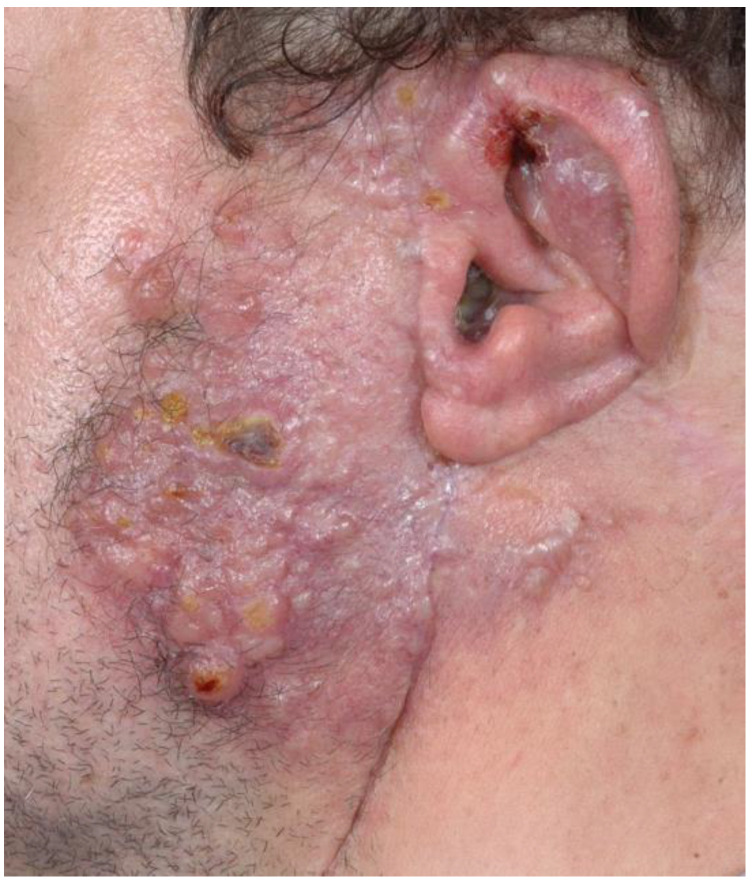
Recurrent EHE in the region of the left cheek, parotid gland and auricle after previous surgery and radiation therapy.

The clinical course of EHE is highly variable and depends on its localization (Figure 2).

EHE is often incidentally diagnosed, and over 50–76% of patients are asymptomatic [66]. Typically, the lesion presents in patients between the age of 30 and 50 years [60,67]. In an effort to reflect different biologic subsets, Deyrup et al. suggested classifying EHE into two risk groups, with markedly different clinical courses depending on tumor size and mitotic activity [68]. High risk was defined as having a tumor size >3.0 cm and >3 mitotic figures/50 high power fields. Neither of these characteristics was considered as low risk. Patients with high-risk tumors had a 5-year disease-specific survival of 59%; no patients with low-risk tumors died. Metastatic rates were 15% and 32% in low-risk and high-risk patients, respectively [68].

### 3.3. Histology

Macroscopically, EHE are nodular or multinodular, with a pale firm cut surface with variable, commonly subtle hemorrhage [57]. Histologically, EHE is characterized by cords, strands or small nests of large endothelial cells, histiocytoid and/or spindled cells with abundant eosinophilic, often vacuolated cytoplasm embedded in a myxohyaline stroma. It has round, vesicular, occasionally intended nuclei. Atypical nuclei, pleomorphism and mitotic figures can be observed. The presence of necrosis and higher numbers of fusiform neoplastic cells may suggest a more aggressive clinical course, including distant metastasis. Immunostaining indicates that EHE is positive for CD31, CD34, factor VIII-related antigens, FLI1, other endothelial markers, and the lymphatic endothelial marker podoplanin. Immunostaining of cytokeratin and endothelial markers such as CD31, CD34 and factor VIII can also be used to differentiate EHE from carcinomas. Approximately 25% of EHE show immunoreactivity to cytokeratins, and 45% react to smooth muscle actin. Some studies have proposed the use of podoplanin as an immunohistochemical marker in order to differentiate EHE from nonvascular tumors [69].

### 3.4. Staging

Deciding on the appropriate therapy for patients with EHE mandates accurate CT or MRI tumor staging with whole-body coverage, including the brain. CT scans of the chest, abdomen and pelvis should be performed due to their wide coverage and optimal assessment of pulmonary disease. Based on its high spatial resolution and soft tissue contrast, MRI seems well suited for primary soft tissue disease, parenchymal and osseous lesions. Most frequently, EHE shows low to intermediate signal intensity on T1-weighted MRI and high signal intensity on T2-weighted MRI, together with homogeneous enhancement after the injection of gadolinium. A bone scan should be performed in order to exclude bone lesions. Upon availability, a (FDG)-PET/CT can also be done. Usually, the FDG uptake is mild to moderate. Previous studies have demonstrated that higher SUV_max_, SUV_peak_, TLG and MTV of lesions in (FDG)-PET, indicate a worse prognosis [70,71].

### 3.5. Therapy

Because of its rarity, there is no standard treatment for EHE. Indeed, few therapeutic options are available. There are only case reports and case series, and no systematic trials for EHE in the head and neck area (Table 3).

Surgery is the treatment of choice for localized lesions. In the head and neck area, extended surgery can be associated with high functional impairments and aesthetic deformity, and should therefore be carefully considered based on the location of the lesion and morbidity of the radical approach. In case of sinunasal tumors, endoscopic and 3D endoscopic approaches are feasible. In particular, 3D endoscopes offer optimal vision and additional information about depth, anatomical landmarks and details as well as better orientation in the surgical field [96]. In tumors located in the pharynx and larynx, transoral surgery is a therapeutic option providing the benefit of less morbidity, fewer complications and shorter surgical times. Especially in tumors of the tongue base, transoral robotic surgery can be performed, which leads to improved visualization with three-dimensional imaging and the advantages of angled scopes, tremor filter, and improved range of motion [97,98]. In patients with suspected local residues or for whom surgery is impossible, radiotherapy should be considered as a therapeutic option [88].

Indication for radiation depends primarily on the resectability of the tumor and the risk of recurrence, which is estimated to be 10–15% after complete surgical resection. Radiotherapy in EHE is based on the principles of soft tissue sarcoma treatment. Studies suggests EHE to be a reasonably radiosensitive tumor, showing response to a dose of 30–40 Gy [99]. In selected cases with positive or close resection margins, adjuvant RT with a total dose of 60 Gy in 30 fractions may be advised, as it has been shown to be effective in maintaining local control. For patients with inoperable EHE, definitive RT with a total dose in the order of 60 Gy in conventional fractionation is recommended. Preoperative radiotherapy for EHE has not yet been evaluated; analogous to the treatment of soft tissue sarcomas, therapy with 50 Gy in 25 fractions may possibly be used in individual cases. For symptom relief in cases with metastases, 30–60 Gy may be sufficient depending on the affected anatomical site.

In asymptomatic patients with diffuse lesions, watchful waiting may be performed in selected individual cases, as spontaneous regressions have been reported in patients with pulmonary EHE [100].

Systemic treatment should only be performed in symptomatic patients with metastatic disease [69]. In asymptomatic patients with advanced metastatic EHE without the possibility of resection with acceptable morbidity, active surveillance is the preferred approach [69]. In patients with localized, resectable EHE, there is no evidence for the effectiveness of systemic neoadjuvant or adjuvant therapy. In metastatic disease, different systemic treatment methods have been evaluated including cytotoxic chemotherapy, immune therapy, and targeted therapies. As conventional chemotherapy appears to have very limited efficacy in EHE patients, systemic therapy should be limited to aggressive or rapidly progressive cases. A standard systemic therapy has not been established so far.

In case reports and small case series, cytotoxic chemotherapy has been explored in EHE patients. The regimens included carboplatin, paclitaxel, adriamycin, dacarbazine, and ifosfamide [39,69,101]. In all, these studies scarcely include patients with primary EHE in the head and neck region. Response rates to cytotoxic chemotherapy were rather bad. Anthracycline-based chemotherapy, which is a standard first-line treatment in soft tissue sarcoma, has demonstrated only very limited or no effect in EHE patients [101]. Patients with EHE also did not benefit from paclitaxel, which is often used in sarcoma treatment. Pazopanib showed some effect in two retrospective case series [31,101], while another study did not show any benefit, with a PFS of 2.9 months [101].

The mTOR inhibitor sirolimus has also been evaluated in EHE, leading to prolonged stabilization in most patients who present without serosal effusions. In one study, patients with advanced and progressing EHE were treated with sirolimus 5 mg daily until they developed either toxicity or disease progression. Disease progression in the 6 months before the start of treatment was required. In all, four patients (10.8%) showed a partial response, stable disease was seen in 28 patients (75.7%), and disease progression in five patients (13.5%). The median PFS was 13 months, and the median OS was 18.8 months at a 41.5-month median follow-up. Serosal effusions were confirmed as an unfavorable prognostic sign associated with short survival, and sirolimus displayed limited activity in this subgroup [102].

Therapies targeting VEGFR2 have also been described for EHE. In case reports, Apatinib and Sorafenib led to clinical and radiographic response [103,104]. In a patient with metastatic EHE of the cervical and mediastinal lymph nodes, lungs, and liver, pazopanib also led to partial response [105].

Given the vascular origin of EHE, the use of antiangiogenetic drugs such as thalidomide and lenalidomide reasonably might be effective. Until now, data on the use of thalidomide in the treatment of EHE have been limited to a small number of case reports demonstrating clinical benefits for its use either as monotherapy [106] or in combination with other anti-angiogenic agents [107]. Studies on larger patient groups are still pending. Case reports have also demonstrated some antitumor activity of interferon [108,109]; however, the real benefit remains unclear.

## 4. Conclusions

Surgery remains the mainstay of treatment for malignant vascular tumors of the head and neck. The role of radiotherapy is not clearly defined at present, although it is used as adjuvant treatment in many cases. A standard medical therapy for metastasized malignant vascular tumors is lacking. Chemotherapy, which is effective in angiosarcoma, is mostly ineffective in EHE. Due to the rarity of these diseases, prospective trials are difficult; however, EHE and angiosarcoma patients should be considered for clinical trials when available, and should be managed within sarcoma reference centers or reference networks by a dedicated sarcoma multidisciplinary team. In the age of targeted therapies and incorporation of next generation sequencing into treatment strategies, there should be great interest in identifying potentially targetable genomic alterations in order to personalize treatment approaches, especially for these rare tumors.

## Figures and Tables

**Table 1:** Classification of vascular tumors according to the ISSVA 2018 [1].

Benign	Locally Aggressive or Borderline	Malignant
Infantile hemangioma/Hemangioma of infancy Congenital hemangioma Tufted angioma Spindle-cell hemangioma Epithelioid hemangioma Pyogenic granuloma	Kaposiform hemangioendothelioma Retiform hemangioendothelioma Papillary intralymphatic angioendothelioma Composite hemangioendothelioma Pseudomyogenic hemangioendothelioma Polymorphous hemangioendothelioma Hemangioendothelioma not otherwise specified Kaposi sarcoma	Angiosarcoma Epithelioid hemangioendothelioma
Others: Hobnail hemangioma Microvenular hemangioma Anastomosing hemangioma Glomeruloid hemangioma Papillary hemangioma Intravascular papillary endothelial hyperplasia Cutaneous epithelioid angiomatous nodule Acquired elastotic hemangioma Littoral cell hemangioma of the spleen		
Related lesions: Eccrine angiomatous hamartoma Reactive angioendotheliomatosis Bacillary angiomatosis		

**Table 2:** Trials on systemic treatment in angiosarcoma patients.

Author, Year [Reference]	Treatment	Number of Patients	ORR	Median PFS (Months)	Median OS (Months)
Fata 1999 [24]	paclitaxel	9	89%	5	NR
Butt 2002 [25]	doxorubicin	33	33%	NR	NR
Nagano 2002 [26]	docetaxel	39	67%	9.5	NR
Fury 2005 [27]	Doxorubicin paclitaxel	30 41	NR NR	3.7–5.4 4	NR NR
Schlemmer 2008 [16]	paclitaxel	32	63%	7.6	NR
Ryan 2008 [28]	sorafenib	9	11%	4.7	13.5
George 2009 [29]	sunitinib	2	0%	NR	NR
Maki 2009 [30]	sorafenib	37	14%	3.8	14.9
Penel 2012 [31]	paclitaxel doxorubicin different chemotherapies	47 70 16	45.5 30.9 12.3	5.6 3.9 3.2	13.1 11 9.7
Stacchiotti 2012 [32]	gemcitabine	25	64	7	17
Ray-Coquard 2012 [33]	sorafenib	41	14.6	2	9.7
Italiano 2012 [34]	paclitaxel doxorubicin	75 42	53 29	5.8 3	10.3 5.5
Agulnik 2013 [35]	bevacizumab	23	9	3	11
Young 2014 [36]	anthracycline based	108	25	4.9	NR
DÁngelo 2015 [37]	anthracycline-based taxane-based other agents	74 74 30	25–33 31 NR	3.4 3.6 3.0	12 11.6 17.8
Ray-Coquard 2015 [38]	paclitaxel paclitaxel+ bevacizumab	26 24	45.8 28.5	6.6 6.6	19.5 15.9
Kollar 2017 [39]	pazopanib	40	20	3	9.9
Lebellec 2018 [40]	paclitaxel paclitaxel+ bevacizumab	18 24	NR NR	5.5 5.7	NR NR
Agulnik 2021 [41]	regorafenib	23	17.4	5.5	NR
Wagner 2021 [42]	nivolumab+ ipilimumab	16	25	NR (6 months PFS: 38%)	NR

ORR: objective response rate; PFS: progression-free survival; OS: overall survival; NR: not reported.

**Table 3:** Cases of EHE in the head and neck area.

Author Year	Gender	Age	Localization	Clinical Presentation	Metastasis at Diagnosis	Initial Therapy	Recurrence	Therapy at Recurrence	Follow-Up
Siddiqui 1998 [72]	f	44	thyroid	local neck discomfort, gradual increase in size of mass, weakness, hoarseness	no	resection	no	no	2 years: NED
Hassan 2005 [73]	f	73	thyroid	mass, hoarseness, dysphagia, weight loss	no	resection	local recurrence at 9 months	palliative surgery, 2 months of subcutaneous interferon-alpha therapy	died 13 months after diagnosis
Naqvi 2008 [74]	m	4	nasal cavity	NR	no	resection	local recurrence after 3 and 5 years	resection (2×)	10 years: NED
	f	17	gingiva	NR	no	resection, radio- and chemotherapy	local recurrence after 4 years; local recurrence+ lymph node metastases after 5 years.	resection NR	NR
	f	66	gingiva	NR	lymph node metastasis	resection lymph node dissection	no	NA	10 months: NED
	m	71	tongue	NR	no	resection	NR	NR	NR
Wong 2009 [75]	m	50	forehead	skin lesion, visual disturbance	no	resection	NR	NR	NR
Patnayak 2010 [76]	m	40	nasal cavity	swelling, intermittent epistaxis	no	resection	NR	NR	9 months: NED
Al-Faky 2011 [77]	f	27	eyelid	mass	no	resection	no	NA	2 years: NED
Banerjee 2013 [78]	f	30	neck	huge neck swelling	no	resection	NR	NR	6 months: NED
Drazin 2013 [79]	m	62	mastoid	dizziness, nausea	no	resection	local recurrence after 15 months	resection, radiotherapy 59.4 Gy	8 years: NED
Ma 2013 [80]	f	58	clivus	headache, visual detoriation	no	resection	NR	NR	NR
Ali 2015 [81]	f	23	gingiva	swelling	no	resection	local recurrence after 7 years	resection	16 years: NED
Shah 2016 [82]	f	35	thyroid	local neck discomfort, sore throat, hoarseness, dysphagia, weight loss	no	resection, neck dissection	residual disease	radio-chemotherapy	NR
Hanege 2016 [83]	m	62	nasal septum	epistaxis, congestion	no	resection	no	NA	3 years: NED
Sancheti 2016 [84]	f	25	hypopharynx	dysphagia, difficulties in breathing	no	resection	no	NA	1 year: NED
Ogita 2016 [85]	f	27	nasal cavity	epistaxis, pain	no	resection	no	NA	2 years: NED
Salgarelli 2016 [86]	m	33	mandibular gingiva	lesion	no	resection	3 neck lymph node metastases 4 years later	resection	NR
Brill 2016 [87]	f	39	mediastinum	mass	no	resection	no	NA	1 year: NED
Duzer 2017 [88]	f	26	neck	lump in the neck	no	resection, postoperative radiotherapy	NR	NR	NR
Koide 2018 [89]	f	70	parotid gland	swelling, pain	no	resection, neck dissection, adjuvant radiation 60 Gy	distant metastases (right lung, lumbar spine, liver) 5 months after surgery	no	died 13 months after diagnosis
Ennouhi 2018 [90]	NR	NR	eyelid	mass	no	resection	no	NA	5 years: NED
Jamshidian-Tehrani 2019 [91]	m	30	orbit	proptosis, hypoglobus	no	resection	NR	NR	NR
Suarez-Zamora 2019 [92]	f	62	parotid gland	painless mass	no	resection	no	NA	NR
Komatsu 2020 [93]	m	66	gingiva	gingival swelling	no	resection, paclitaxel	no	NA	1 year: NED
Lui 2021 [94]	male	52	vocal fold	dysphonia	no	radiation therapy (5000 cGy)	multiple pulmonary metastases at one year	NR	NR (metastases at one year)
Cirkin 2021 [95]	m	55	tongue	lumps in the tongue, pain	no	resection	NR	NR	NR

NR: not reported; NA: not applicable; NED: no evidence of disease.
